# Artificial Intelligence in emergency department triage: A scoping review

**DOI:** 10.1371/journal.pone.0352338

**Published:** 2026-06-25

**Authors:** Laura Lima Souza, Yasmim Carolaine Nascimento de Oliveira, Luzia Clênia Campos da Costa, José Aguinaldo Alves da Silva Filho, Ana Tereza Freire de Souza, Vanessa Gomes Mourão, Rodrigo Assis Neves Dantas, Fabiane Rocha Botarelli, Kátia Regina Barros Ribeiro

**Affiliations:** 1 Graduate Program in Nursing, Universidade Federal do Rio Grande do Norte, Natal, Rio Grande do Norte, Brazil; 2 Nursing Program, Nursing Department, Universidade Federal do Rio Grande do Norte, Natal, Rio Grande do Norte, Brazil; University of Porto Faculty of Medicine: Universidade do Porto Faculdade de Medicina, PORTUGAL

## Abstract

**Background:**

Triage in emergency departments (ED) is a critical process for prioritizing care and ensuring clinical safety. However, current triage systems often exhibit vulnerabilities that compromise the efficiency and quality of healthcare delivery. Artificial Intelligence (AI) has emerged as a promising innovation to support decision-making and optimize patient flow in these high-pressure environments.

**Objective:**

To map the available evidence regarding the implementation and performance of artificial intelligence in emergency department triage.

**Method:**

This scoping review followed the Joanna Briggs Institute (JBI) methodology and the PRISMA-ScR guidelines. A comprehensive search was conducted across 13 databases (CINAHL, Cochrane Library, PubMed Central, SciELO, Web of Science, SCOPUS, Science Direct, VHL, Embase, and several regional dissertation repositories), with no language or time restrictions. Two independent reviewers performed the selection process using the Rayyan platform, with discrepancies resolved by a third evaluator. Data were synthesized using the PAGER framework, categorizing findings into Patterns, Advances, Gaps, Evidence for practice, and Recommendations for research.

**Results:**

Nineteen studies met the inclusion criteria. AI was primarily implemented through Machine Learning (ML) algorithms, including Deep Learning architectures. Natural Language Processing (NLP) was frequently employed to process unstructured clinical data, with recent studies exploring the potential of Large Language Models (LLMs). Overall, ML-based models consistently outperformed traditional triage systems in predictive accuracy. These techniques were mainly utilized for automated classification, predicting clinical severity, and enhancing patient prioritization by integrating both objective and subjective assessment data.

**Conclusions:**

The findings indicate that AI has significant potential to enhance emergency triage by streamlining service flows and providing robust clinical decision support. However, the current evidence remains heterogeneous and largely exploratory. Key challenges include variability in model performance, a lack of external validation, and studies often limited to specific populations. Consequently, many current tools still lack the necessary reliability for safe, large-scale clinical implementation.

## 1. Introduction

Hospital Emergency Services serve as critical entry points into healthcare systems. These departments frequently face high demand leading to overcrowding, which can significantly compromise clinical outcomes [1]. Globally, “triage” refers to the systematic prioritization of patients based on clinical severity to organize patient flow and allocate resources efficiently. In many healthcare settings, this process is also termed “risk classification”; both terms describe the same structured clinical assessment used to determine urgency. In this study, “triage” and “risk classification” are used interchangeably. This process remains a cornerstone of emergency care, essential for prioritizing critical patients, enhancing safety, and optimizing service delivery [2,3].

However, the effectiveness of triage is often hindered by human factors. Variability in professional experience, clinical competencies, and individual characteristics can lead to process failures, such as incorrect patient categorization, inaccurate documentation, and diagnostic errors—all of which directly impact patient safety [4]. Among established protocols, the Manchester Triage System (MTS) is widely adopted, categorizing patients into urgency levels ranging from emergent to non-urgent [2,5]. Nevertheless, the isolated implementation of the MTS does not guarantee optimal care; it requires continuous monitoring and managerial refinement [2]. Consequently, nurses must continuously develop advanced clinical assessment skills, rapid decision-making abilities, and active listening to ensure swift risk identification and appropriate care pathways [1,6].

To address challenges in accuracy and agility, various technologies have been integrated into emergency services to streamline initial care. Among these, Artificial Intelligence (AI) has emerged as a transformative tool, capable of processing vast clinical datasets, recognizing complex patterns, and providing real-time decision support by simulating human perception and analytical reasoning [7].

Machine Learning (ML), a specialized subfield of AI, offers significant potential for the early detection of clinical conditions and outcomes within the emergency department. Furthermore, ML facilitates safer and more efficient referrals through the sophisticated analysis of electronic health records (EHR) [8,9]. Research indicates that triage precision increases when nurses utilize technological clinical decision support systems (CDSS), fostering greater safety and reducing adverse events [10,11]. Thus, adopting ML as a complementary resource to traditional risk assessment enhances service quality by providing evidence-based, rapid insights [7,8].

The development of ML models typically follows a structured pipeline: data acquisition, pre-processing, model training (where algorithms learn patterns from historical data), and validation (testing the model on unseen data to assess predictive performance) [8]. Within clinical contexts, two concepts are paramount: algorithmic bias, which occurs when training data reflects historical inequalities or poor data quality, leading to unfair or erroneous predictions [8]; and interpretability, the ability of healthcare professionals to understand the logic behind a model’s output [7,8]. Ultimately, the successful integration of these tools into clinical practice depends on their capacity to function as effective decision support systems that improve accuracy without compromising patient safety [10,11].

Given this context, it is essential to analyze how AI has been integrated into triage and risk classification processes. Synthesizing the available evidence and identifying knowledge gaps is crucial to guiding future research and enhancing clinical practice. Therefore, this study aims to map the scientific evidence regarding the application of AI in triage and risk classification within Emergency Services.

## 2. Method

### 2.1. Study design

This scoping review aims to map the scientific evidence regarding the application of AI in triage and risk classification within emergency services. The study was conducted following the methodological framework proposed by the Joanna Briggs Institute (JBI) [12] and adheres to the Preferred Reporting Items for Systematic Reviews and Meta-Analyses Extension for Scoping Reviews (PRISMA-ScR) checklist [13] (see S1 File). The alignment with these frameworks ensures transparency, reproducibility, and methodological rigor across all research stages [14].

The PCC strategy (Population, Concept, and Context) was employed to formulate the research question: Population (P): Patients undergoing risk classification/triage; Concept (C): Artificial Intelligence; and Context (C): Hospital Emergency Services. Thus, the research question was: How has AI been utilized in risk classification and triage within emergency services?

To ensure originality and prevent duplication, a preliminary search for registered reviews was conducted in the International Prospective Register of Systematic Reviews (PROSPERO), Open Science Framework (OSF), The Cochrane Library, and the Database of Abstracts of Reviews of Effects (DARE). No reviews with an identical thematic focus were identified. Consequently, this study protocol was registered on the Open Science Framework (osf.io/z2hu7; https://doi.org/10.17605/OSF.IO/Z2HU7).

### 2.2. Selection process and search strategy

The literature search was performed across 13 databases: CINAHL, Cochrane Library, PubMed Central, SciELO, Web of Science, SCOPUS, Science Direct, the Virtual Health Library (VHL), and Embase. Grey literature sources included the CAPES Thesis and Dissertation Catalog, the Brazilian Digital Library of Theses and Dissertations (BDTD), the Scientific Open Access Repository of Portugal (RCAAP), and Theses Canada. Data collection was finalized in September 2025. Access to these sources was facilitated via the CAPES Journal Portal through the Federated Academic Community (CAFe) platform.

Search terms were identified using Health Sciences Descriptors (DeCS) and Medical Subject Headings (MeSH), with adaptations for English and Portuguese. The primary descriptors included: “Risk Classification”, “Risk Assessment”, “Artificial Intelligence”, and “Emergency Service, Hospital”.

The selection of these descriptors was a strategic decision to enhance search specificity. During the pre-analytical phase, sensitivity tests using broader terms—such as “Triage” or “Emergency Department”—yielded an excessive volume of non-relevant results (low precision), primarily related to manual protocols. To maintain methodological rigor, more targeted descriptors (“Risk Classification” and “Risk Assessment”) were combined with “Artificial Intelligence”. This strategy ensured the identification of studies strictly aligned with AI-driven structured risk classification.

Boolean operators “AND” and “OR” were used to combine descriptors, adapted to the syntax requirements of each database without temporal or language restrictions. The core search string was: (“Risk Classification” OR “Risk Assessment”) AND “Artificial Intelligence” AND “Emergency Service, Hospital”. Detailed search syntaxes for each database are provided in Table 1.

**Table 1:** Search strategy and syntax applied to each database.

Data source	Search syntax
Cumulative Index to Nursing and Allied Health Literature (CINAHL)	((Risk Assessment OR Risk Classification) AND (Artificial Intelligence) AND (Emergency Service, Hospital))
Cochrane Library	(“Risk Assessment”) in All Text OR (“Risk Classification”) in All Text AND (“Artificial Intelligence”) in All Text AND (“Emergency Service, Hospital”)
PubMed Central	((Risk Assessment OR Risk Classification) AND (Artificial Intelligence)) AND (Emergency Service, Hospital))
Scielo	(Risk Assessment OR Risk Classification) AND (Artificial Intelligence) AND (Emergency Service, Hospital)
Web of Science	((TS=((Risk Assessment OR Risk Classification))) AND TS=((Artificial Intelligence))) AND TS=((Emergency Service, Hospital))
SCOPUS	(TITLE-ABS-KEY (“Risk Assessment” OR “Risk Classification”) AND TITLE-ABS-KEY (“Artificial Intelligence”) AND TITLE-ABS-KEY (“Emergency Service, Hospital”))
Science Direct	(Risk Assessment OR Risk Classification) AND (Artificial Intelligence) AND (Emergency Service, Hospital)
CAPES Thesis and Dissertation Catalog	(Medição de risco OR Classificação de risco) AND (Inteligência Artificial) AND (Serviços de Emergência)
Virtual Health Library (BVS)	(Medição de risco OR Classificação de risco) AND (Inteligência Artificial) AND (Serviços de Emergência)
Embase	(‘risk assessment’ OR ‘risk classification’) AND ‘artificial intelligence’ AND ‘hospital emergency service’
Brazilian Digital Library of Theses and Dissertations (BDTD)	(“Medição de risco “ OR “Classificação de risco”) AND (“Inteligência artificial”) AND (“Serviços de Emergência”))
Scientific Open Access Repository of Portugal (RCAAP)	(“Risk Assessment” OR “Risk Classification”) AND (“Artificial Intelligence”) AND (“Emergency Service, Hospital”)
Theses Canada	(“Risk Assessment” OR “Risk Classification”) AND (“Artificial Intelligence”) AND (“Emergency Service, Hospital”)

### 2.3. Eligibility criteria

Studies were included if they addressed the application of AI in triage or risk classification within emergency services. The inclusion criteria encompassed original research articles, dissertations, theses, ministerial ordinances, and clinical guidelines. To be eligible, studies had to be available in open access or in full text through the café platform. Conversely, studies were excluded if they did not directly answer the research question, or if they were abstracts, reviews, experience reports, letters to the editor, or book chapters

### 2.4. Selection and data extraction

The literature search, screening, and selection processes were conducted independently and concurrently by two reviewers using separate electronic devices, following a structured four-stage workflow. Initially, duplicate studies were identified and removed using the Rayyan platform, followed by a preliminary screening of titles and abstracts against the established eligibility criteria. Any discrepancies or conflicts during these phases were resolved through consultation with a third reviewer to finalize the selection. In the final stage, the remaining studies underwent a comprehensive full-text review to determine their definitive inclusion in the final sample.

For data extraction, a standardized Microsoft Word table was used to record the following variables: author, year of publication, country of origin, objectives, methodology, sample size/characteristics, keywords, and primary findings. Following the primary selection, a reverse search (reference chaining) was performed. The bibliographies of all included studies were manually screened to identify additional relevant research that met the inclusion criteria.

### 2.5. Synthesis of results and framework

The results are presented through flowcharts and tables, highlighting variables directly aligned with the research objective. To ensure a structured and comprehensive analysis, data synthesis was guided by the PAGER framework (Patterns, Advances, Gaps, Evidence for practice, and Research recommendations) [15].

### 2.6. Ethical considerations

As this study is a scoping review of publicly available literature and did not involve human participants, formal ethical approval was not required.

## 3. Results

### 3.1. Study selection

**Fig 1:**
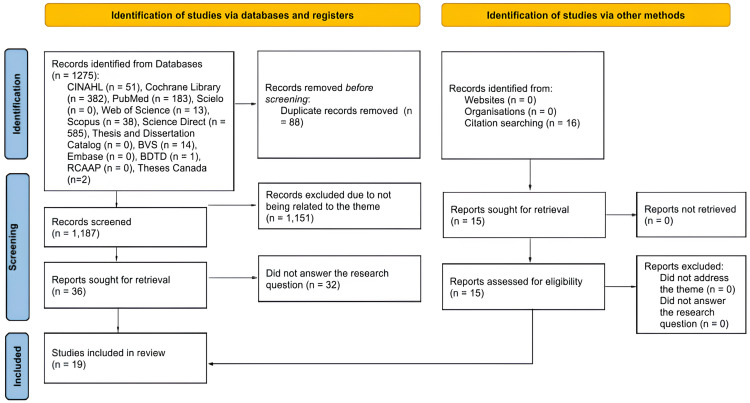
Synthesis of the literature mapping process (Adapted from Prisma). ¹ Flowchart adapted from PRISMA-ScR.

The initial search yielded 1,275 records across all data sources. After removing duplicates, 1,187 titles and abstracts were screened, of which 1,151 did not meet the eligibility criteria. Consequently, 36 full-text articles were assessed for eligibility. Of these, 32 were excluded for not directly addressing the research question. To ensure a comprehensive mapping, a reverse search (reference chaining) was performed on the remaining studies, identifying 15 additional relevant articles. The final sample comprised 19 studies, as detailed in the PRISMA-ScR flowchart (Fig 1).

### 3.2. Characteristics of the included studies

The included studies were published between 2013 and 2025, with a notable increase in recent years. The highest concentration occurred in 2021 (n = 4; 21%) and 2023 (n = 3; 16%), followed by a steady output in 2019, 2022, 2024, and 2025 (n = 2 each; 11%). Earlier contributions were sporadic, with single publications (5% each) in 2013, 2016, 2018, and 2020.

Geographically, research was predominantly conducted in Asia (n = 9; 47%) and Europe (n = 7; 37%), followed by North America (n = 2; 11%) and Latin America (n = 1; 5%). Regarding study design, observational frameworks prevailed: cross-sectional (n = 7; 36.8%) and retrospective (n = 7; 36.8%) designs were most frequent. The remaining sample included systematic reviews (n = 3; 15.8%), predictive modeling studies (n = 1; 5.3%), and narrative reviews (n = 1; 5.3%).

The professional profile of the authors was multidisciplinary, although predominantly medical (65.2%), with a focus on emergency medicine. Nursing professionals accounted for 6.5% of the authorship. Significant contributions also came from engineering fields, including data science (6.5%), computer science (4.3%), and electrical, electronic, and industrial engineering (combined 5.4%). Other collaborators included specialists in public health (8.1%), epidemiology, statistics, biomedicine, and informatics (1% each).

### 3.3. AI approaches and architectures

All identified AI applications fell within the broad category of machine learning (ML), as well as its subfield, deep learning (DL). This paradigm enables machines to be trained to process information, make decisions, and solve problems based on data that forms patterns. Traditional ML algorithms—such as Logistic Regression and Gradient Boosting—were frequently used. In addition, Federated Learning stood out, characterized by the training of models using decentralized data. Several studies also implemented more complex DL architectures, particularly Artificial Neural Networks (ANNs) and Deep Neural Networks (DNNs), which were sometimes integrated with neuro-fuzzy systems.

In terms of application domains, Natural Language Processing (NLP) was a recurring theme for processing unstructured textual and speech data. Within this domain, recent studies (n = 2; 10%) evaluated the performance of Large Language Models (LLMs), specifically ChatGPT. Only one study (5%) described an AI-based clinical decision support system without explicitly specifying the underlying algorithm. Detailed characteristics of the included studies are presented in Table 2.

**Table 2:** Characteristics of included studies.

ID/ YEAR/ COUNTRY	OBJECTIVES	METHODS	MAIN RESULTS
Porto, B.M. (2024)/ Brazil [16]	Systematically review studies that employed ML and/or Natural Language Processing methods to classify adult and pediatric patient triage in EDs [16].	Systematic Review. Sample: 60 studies analyzed. AI subfield: ML and Natural Language Processing [16].	Logistic regression was the most used model. These models outperformed the Korean Triage Acuity Scale (KTAS) and Emergency Severity Index (ESI) systems in adults and children. ML and NLP would be applied as a complementary approach to traditional methods [16].
Sánchez-Salmerón, R. et al. (2022)/ Spain [17]	Analyze the effectiveness of ML systems in triage for making predictions in the emergency department compared to other triage scales/scores [17].	Systematic Review. Sample: 124 studies analyzed, 11 included. AI subfield: ML [17].	Studies show that ML models improve the prediction of cardiovascular events, mortality, need for intensive care or hospitalization, and high-risk disease diagnoses compared to other conventional scales [17].
Chang, H. et al. (2023)/ South Korea [18]	This study developed a Federated Learning-based Clinical Support System to predict the need for a revised Korean Triage Acuity Scale to facilitate triage [18].	Retrospective Observational Study. Sample: Patients visiting 151 emergency departments. AI subfield: Federated Learning [18].	The system used the initial KTAS at triage for revision and prediction. Improvements in the accuracy of the acuity levels were identified; thus, this model can assist in identifying patients who will require acuity revision, reducing errors with decision-making support [18].
Choi, S.W. et al. (2019)/ South Korea [19]	Train and compare ML models on their ability to predict KTAS levels [19].	Cross-sectional Study. Sample: Tertiary University Hospital Emergency Department. AI subfield: ML and Natural Language Processing [19].	The addition of textual data can predict KTAS levels with high performance during emergency room triage. Textual triage data improved prediction performance in all studied models [19].
Jiang, H. et al. (2021)/ China [20]	Train and compare the performance of four common ML models to assist in decision-making on triage levels [20].	Cross-sectional Study. Sample: 17,661 patients with cardiovascular disease (CVD). AI subfield: ML for decision support [20].	ML models exhibited moderate performance and capability to accurately classify patients with suspected cardiovascular diseases. In addition to having a good aptitude for identifying, among MTS/ESI level 3 patients, those who will require Intensive Care Unit admission [20].
Liu, Y. et al. (2021)/ China [21].	Derive and validate a ML method to support the identification of potentially fatal incorrect triage and offer a detailed real-time explanation at triage [21].	Cross-sectional Study. Sample: 22,272 included cases (database analysis). AI subfield: ML Models [21].	The method proved efficient in the precision of recognizing high-risk patients, reducing the rate of life-threatening incorrect triage and modifying the clinical behavior of the triage professional team [21].
Gao, Z. et al. (2021)/ China [22].	Establish an emergency triage model through statistical analysis of big data over a specific period from a hospital information system to improve triage accuracy in the emergency department [22].	Predictive Observational Study. Sample: 276,164 patients. AI subfield: ML Models [22].	The model applied to emergency classification can improve human resource allocation and optimize patient diagnosis and treatment, thus reducing overtriage and undertriage [22].
Joseph, J.W. et al. (2020)/ United States [23].	Examine whether DL approaches could identify critically ill patients using only data immediately available at triage [23].	Cross-sectional and Retrospective Study. Sample: 445,925 adult patients. AI subfield: Deep Neural Network (DNN) Model [23].	The neural network-based model applied at triage was able to capture subtle indicators of patient severity through the analysis of vital signs and main complaints. It also showed higher precision in identifying critical clients compared to traditional models [23].
Levin, S. et al. (2018)/ United States [24].	Evaluate a ML-based electronic triage (e-triage) system that predicts the probability of acute outcomes, allowing for better patient differentiation [24].	Cross-sectional Study. Sample: 172,726 adult emergency department visits. AI subfield: ML-based tool (e-triage) [24].	The e-triage system was applied in initial care, using electronic health record data to identify patterns of clinical severity. Compared to the ESI, it achieved equal or superior performance in predicting higher risk for mortality, intensive care unit admission, and emergency procedures [24].
Kim, D. et al. (2021)/ South Korea [25].	Develop an automatic triage system using speech recognition and natural language processing for the Korean language to save precious time for doctors in the emergency room [25].	Observational Study. Sample: 762 retrospective cases. AI subfield: Speech Recognition and Natural Language Processing [25].	The NLP-based method was employed to transcribe and interpret patient-professional dialogues, achieving the prediction of the Korean Triage Acuity Scale level and main reported symptoms. The BERT-KTAS model showed good generalization potential and robust results [25].
Zaboli, A. et al. (2024)/ Italy [26].	Compare the accuracy in risk classification between human healthcare professionals and the ChatGPT language model, using the Manchester Triage System [26].	Comparative Observational Cross-sectional Study. Sample: 30 clinical cases. AI subfield: Open-access natural language [26].	Chat-GPT is not particularly effective in stratifying admitted patients at triage when requested, nor is it reliably capable of using the Manchester Triage System. The current level of reliability is insufficient for it to serve as a substitute for professional knowledge in the emergency triage setting [26].
Sarbay, I. et al. (2023)/ Turkey [27].	Determine the performance of ChatGPT in predicting triage in emergency medicine (EM) [27].	Preliminary and Cross-sectional Study. Sample: 50 triage case scenarios. AI subfield: Open-access natural language (ChatGPT) [27].	ChatGPT performed poorly in ESI triage categories based on clinical description but showed better results in high-acuity distinction, achieving good sensitivity and specificity. The method can contribute to initial triage for classifying patients at imminent risk [27].
Gao, F. et al. (2022)/ France [28].	Evaluate the performance of recently described ML models for patient triage in emergency departments and identify future challenges [28].	Systematic Review. Sample: 37 Studies analyzed. AI subfield: ML and Natural Language Processing Models [28].	The analyzed studies presented major methodological flaws, however, the review highlights that the inclusion of textual data through Natural Language Processing significantly improved the models, thus potentially contributing to triage in identifying high-risk patients [28].
Azeez, D. et al. (2013)/ Malaysia [29].	Develop an intelligent triage system with minimal human intervention from specialists in an emergency department [29].	Retrospective Observational Study. Sample: 2223 samples. AI subfield: Neuro-fuzzy System and Artificial Neural Network Model [29].	The comparison of the two models for predicting triage categories in the emergency room showed that the Artificial Neural Network achieved better performance, being more precise, sensitive, and having a lower error rate [29].
Vântu, A. et al. (2023)/ Romania [30].	Research supervised ML models applied to a multiclass classification problem, such as predicting an emergency code for an individual in the specific context of the Romanian emergency medical system [30].	Retrospective Observational Study. Sample: 409 features provided by datasets. AI subfield: ML Models [30].	Supervised models, especially NN-Sequential, can be effective in predicting emergency codes, furthermore in enhancing medical diagnosis and facilitating medical record processing [30].
Zlotnik, A. et al. (2016)/ Spain [31].	Develop and validate a decision support system to predict the probability of hospitalization for patients attended in the emergency department, using the Manchester Triage System and administrative variables routinely collected at the time of triage [31].	Retrospective Observational Study. Sample: The study used a derivation dataset for analysis. AI subfield: Logistic Regression and Artificial Neural Network [31].	The system in question was applied for the prediction of hospital admission for emergency room patients using data obtained after triage via the Manchester Triage System. There was an expansion of the system’s potential to promote more calibrated clinical decisions and optimize team workload [31].
Shafaf, N. et al. (2019)/ Iran [32].	Evaluate the performance of ChatGPT in predicting triage in emergency medicine, using simulated clinical scenarios based on the ESI version 4 [32].	Narrative Review. Sample: Not applicable. AI subfield: ML Models [32].	The results indicate that ML can optimize diagnosis, prognosis, treatment personalization, and data management, however, the authors emphasize that the ethical and safe integration of these technologies, the need for high-quality data, and interdisciplinary collaboration are crucial for its successful implementation in clinical practice [32].
Lindner, G. et al. (2025)/ Austria [33].	Compare the results of patient triage in a large tertiary care emergency department using the Swiss Medical Assessment System (SMASS) alongside the conventional Manchester Triage System [33].	Retrospective Study. Sample: 1021 patients triaged in the service. AI subfield: AI-based decision support system [33].	The use of the system allows for faster triage or even decreases delays in certain stages of patient assessment, in addition to presenting comparable or superior performance to the MST in certain parameters [33].
Da’Costa, A. et al. (2025)/ United Kingdom [34].	Seeks to explore recent advances in AI-based triage systems, focusing on their operational impact in emergency departments and their potential to improve patient outcomes [34].	Narrative Review. Sample: Not applicable. AI subfield: ML [34].	The review identifies substantial benefits of AI-guided triage, including improved patient prioritization, reduced waiting times, and optimized resource allocation [34].

The keywords identified across the studies displayed significant diversity. The term “Triage” was present in all 19 (100%) articles, often associated with “Risk Classification”. “Machine Learning” appeared in 12 (60%) studies, followed by “Emergency Department” (and its variants) in 11 (55%), and “Artificial Intelligence” in 6 (30%). These core concepts strongly align with the descriptors selected for this scoping review.

Regarding the samples, the inclusion methodologies varied significantly. The systematic reviews (n = 3; 15.8%) sampled existing literature based on specific eligibility criteria. Primary studies, including observational (n = 3; 15.8%), cross-sectional (n = 3; 15.8%), and retrospective (n = 1; 5.3%) designs, utilized patient-level data. Other studies (n = 3; 15.8%) focused on large-scale database analysis. Notably, two studies did not specify the exact sample size, delimiting only the clinical sector for data collection. Sample size concepts were considered not applicable to the narrative review and the preliminary/comparative observational studies.

### 3.4. Key findings and model performance

The included research primarily focused on applying ML to enhance the risk classification process. Eight of the 19 studies conducted comparative evaluations against established protocols, with the KTAS and the MTS scales serving as benchmarks in four studies.

Overall, ML-based models consistently outperformed traditional triage methods in terms of predictive accuracy and the identification of high-acuity patients. For instance, Gao et al. reported AUC levels exceeding 0.90 across all severity tiers [22]. Key physiological variables explored included oxygen saturation, systolic/diastolic blood pressure, heart rate, respiratory rate, and biomarkers such as troponin and lactate.

Furthermore, integrating textual data via NLP refined predictive performance in the research by Choi et al. and Kim et al. [19,25]. In contrast, studies evaluating ChatGPT revealed low-to-moderate agreement with traditional systems, suggesting that while LLMs may assist in identifying critical cases, they currently lack the reliability required for independent risk classification [19,20].

To provide a deeper technical overview, Table 3 details the specific algorithms, category, sample sizes, comparators used, external validation and performance metrics. Notably, to address the imbalanced nature of emergency department data, several studies utilized robust performance metrics beyond global accuracy, including Sensitivity, Specificity, Precision, and the Area Under the Receiver Operating Characteristic Curve (AUC).

**Table 3:** Methodological and technical characteristics of the artificial intelligence models in the included studies.

ID	Algorithm	Category	Sample	Comparator used	External validation	Performance metrics
Porto, B.M.	Logistic Regression, XGBoost, Gradient Boosting, Deep Neural Networks (DNN)	ML, DL, NLP	60 studies analyzed.	KTAS, Emergency Severity Index (ESI)	Not applicable	_*
Sánchez-Salmerón, R. et al.	XGBoost, Deep Neural Networks, Random Forest, Logistic Regression	ML, DL	11 studies included.	ESI, KTAS	Not applicable	_*
Chang, H. et al.	Federated Learning (Artificial Neural Network)	ML	Patients visiting 151 emergency departments	KTAS	Present	AUC (Internal): 0.774–0.777; AUC (External): 0.750–0.786; AUPRC: 0.3080.366
Choi, S.W. et al.	XGBoost	ML, NLP	Tertiary University Hospital Emergency Department.	KTAS	Absent	AUC: 0.922; Precision: 0.753; F1-Score: 0.740
Jiang, H. et al.	XGBoost	ML	17,661 patients with cardiovascular disease (CVD).	MTS, ESI	Absent	AUC: 0.937; Accuracy: 0.785
Liu, Y. et al.	CatBoost	ML	22,272 included cases (database analysis)	Team of triage professionals	Absent	AUC: 0.875; Mis-triage rate: 0.9%
Gao, Z. et al.	XGBoost	ML	276.164 patients	Existing protocols of the hospital information system	Absent	Accuracy: 0.825; AUC: 0.912–0.962;
Joseph, J.W. et al.	Deep Neural Network	DL, NLP	445,925 adult patients.	Traditional models	Absent	AUC: 0.857; Sensitivity: 0.845
Levin, S. et al.	Random Forest	ML	172.726	ESI	Absent	AUC (Critical care): 0.90–0.92; AUC (Hospitalization): 0.82–0.84; AUC (Emerg. procedure): 0.73–0.82
Kim, D. et al.	K-Nearest Neighbors (KNN), SVM	ML, NLP	762 retrospective cases.	KTAS and Human Screening	Absent	AUC: 0.890.90
Zaboli, A. et al.	ChatGPT	DL, NLP	30 clinical cases	Human triage nurses using MTS	Absent	Sensitivity: 0.556; Specificity: 0.571; AUC (72-h mortality): 0.669; AUC (Hospital adm.): 0.818; Kappa: 0.278
Sarbay, I. et al.	ChatGPT	DL, NLP	50 triage case scenarios	ESI	Absent	Sensitivity: 0.762; Specificity: 0.931; AUC: 0.846
Gao, F. et al.	Gradient Boosting, Random Forest, Logistic Regression, Deep Neural Networks	ML, DL, NLP	21 analyzed studies	Models that do not use textual data and traditional screening systems.	Not applicable	_*
Azeez, D. et al.	Artificial Neural Network	ML	2.223 samples	Medical officers’ triage	Absent	Accuracy: 96.7%; Sensitivity: 0.82–1.00
Vântu, A. et al.	NN-Sequential	DL	409 features provided by datasets	Models that do not use textual data and traditional screening systems.	Absent	AUC: 0.720.84
Zlotnik, A. et al.	Logistic Regression, Artificial Neural Network	ML, DL	255,668 patients	MST	Absent	AUC (Logistic Regression): 0.857; AUC (Artificial Neural Network): 0.858
Shafaf, N. et al.	Logistic Regression, Support Vector Machine (SVM), Naive Bayes, Decision Tree, Random Forest, Gradient Boosting/ XGBoost, Deep Learning (ANN, LSTM), Bayesian Network, RODDPSO	ML, DL, NLP	Not applicable	ESI	Present	_*
Lindner, G. et al.	Swiss Medical Assessment System, Chatbot	DL, NLP	1.021 patients	MST	Absent	Sensitivity: 0.62; Specificity: 0.73; Kappa: 0.167
Da’Costa, A. et al.	Decision Trees, Neural Networks, NLP models	ML, DL, NLP	Not applicable	Conventional workflow/ Manual sorting systems	Not applicable	_*

**Note:** These records represent review articles (narrative, systematic, or scoping reviews) rather than primary experimental studies. As such, they summarize existing literature and do not report individual primary performance metrics for specific algorithms.

Abbreviations: **ML:** Machine Learning; **DL:** Deep Learning; **NLP:** Natural Language Processing; **ESI:** Emergency Severity Index (5-level triage algorithm); **MTS:** Manchester Triage System (5-priority level protocol); **KTAS:** Korean Triage and Acuity Scale (5-level assessment tool).

### 3.5. Synthesis of evidence (PAGER framework)

The evidence was synthesized using the PAGER framework, categorizing findings into Patterns, Advances, Gaps, Evidence for Practice, and Research Recommendations (Table 4). The analysis indicates that AI is a robust decision-support tool when used as a complementary resource, significantly improving the precision of traditional triage systems.

**Table 4:** Synthesis of findings according to the PAGER framework.

PATTERNS	ADVANCES	GAPS	EVIDENCE FOR PRACTICE	RECOMMENDATIONS FOR RESEARCH
Predominant techniques include Machine Learning (Logistic Regression, Gradient Boosting), Deep Learning (Artificial and Deep Neural Networks), and Natural Language Processing (NLP). Models are frequently benchmarked against established triage scales such as the KTAS, MTS, and other acuity-based protocols.	AI models consistently outperform traditional protocols in identifying high-risk patients and predicting critical outcomes. By leveraging complex datasets, these tools optimize diagnostic accuracy, treatment pathways, and operational efficiency, though performance remains dependent on clinical context and the use of rigorous metrics.	There is a critical deficit in external validation, as most studies are conducted within single institutions and restricted populations, limiting the generalizability of the results. Furthermore, the lack of transparent reporting on accuracy parameters often hinders a comprehensive understanding of model performance.	AI serves as a robust clinical decision support tool, acting as a complementary resource to reduce human error and enhance workflow efficiency in emergency departments. Implementing ML-based systems can significantly optimize patient clinical outcomes and support evidence-based prioritization.	Evaluate the generalizability and long-term outcomes of site-specific ML models, employing advanced statistical modeling to refine risk classification for diverse patient populations.
Accuracy	Accuracy remains the most widely utilized metric to represent the predictive performance of the developed models. Observed values across the studies range from 0.71 to 0.92, indicating a generally satisfactory performance for risk classification tasks.	Despite these high values, the lack of external validation and the use of restricted populations limit the generalizability of the findings. Furthermore, many studies fail to provide a clear explanation of their accuracy parameters or secondary metrics, which complicates a comprehensive understanding of the results in imbalanced clinical settings.	The evidence suggests that current models achieve adequate accuracy to support clinical decisions and contribute significantly to the reduction of clinical judgment errors. This reinforces the potential of AI to enhance the precision and reliability of triage classification.	Future research must prioritize multi-center external validation and the inclusion of diverse populations (e.g., pediatric or elderly). It is essential to contextualize metrics and provide clear explanations of model parameters to ensure transparency and facilitate the integration of site-specific models into broader clinical practice.

## 4 Discussion

### 4.1. Application contexts and AI techniques

The included studies demonstrate a high degree of methodological and contextual diversity, yet they converge on the common objective of evaluating the efficacy of AI models in triage and risk classification. The highest concentration of research was identified in Asia, followed by Europe and North America, with a more limited presence in Latin America. This geographical distribution likely reflects the substantial investments in healthcare technology and the extensive availability of large-scale clinical datasets in high-income regions [35].

Regarding data provenance, the studies leveraged a wide array of sources, ranging from historical literature identified in systematic reviews to real-world patient records extracted from electronic triage systems and emergency department encounters. The use of actual clinical data, as opposed to purely simulated scenarios, enhances the ecological validity of the findings and provides a more robust basis for model validation.

In the analyzed literature, AI was predominantly operationalized through ML and its advanced subfield, DL. While traditional algorithms—such as Logistic Regression and Gradient Boosting—remain prevalent, there is a growing shift toward DL architectures, specifically ANNs and DNNs [16,21–23,29,31]. Furthermore, NLP has emerged as a critical domain for extracting clinical value from unstructured data, such as nursing triage notes. More recently, this has evolved into the evaluation of LLMs, such as ChatGPT, for clinical decision support [19,25–27]. To establish clinical utility, these models were frequently benchmarked against validated triage protocols, including the KTAS, the MTS, and the Emergency Severity Index (ESI) [16,18,20,24,26,33].

A critical observation across the included studies is the limitation of accuracy as a standalone metric. In emergency triage, relying exclusively on accuracy is methodologically misleading due to the inherent class imbalance of patient populations, which are typically skewed toward lower-acuity categories (e.g., ESI/MTS levels 3, 4, or 5). Consequently, an algorithm may achieve high global accuracy by disproportionately predicting low-acuity levels while failing to identify critically ill patients—a phenomenon known as undertriage. To address this, studies focusing on high-stakes outcomes, such as cardiovascular events, ICU admission, or mortality [20,24], expanded their evaluative frameworks to include metrics better suited for imbalanced clinical data, such as sensitivity, specificity, and the Area Under the Receiver Operating Characteristic Curve (AUC). These metrics provide a more reliable detection of critical cases, thereby minimizing life-threatening errors and enhancing patient safety [18,23,27,29]. This performance likely stems from the ability of advanced models, particularly Deep Neural Networks and XGBoost, to capture complex non-linear relationships and subtle patterns within vital signs and medical histories that may be overlooked by traditional, human-led protocols.

### 4.2. Main findings

The literature emphasizes that clinical judgment in triage is inherently susceptible to error, resulting in both overtriage and undertriage. These inaccuracies persist even with validated 5-level instruments due to the high subjectivity and the cognitive load of processing vast amounts of patient data under significant time constraints [24,36]. Such pressures often lead to imprecise classifications and interpersonal conflicts. However, the integration of AI into this workflow has demonstrated the potential to mitigate these errors and enhance overall triage effectiveness [16,20].

Research by Porto [16] confirms that ML and NLP models consistently outperform traditional hospital methods. Beyond superior technical performance, these tools reduce the rates of misclassification and alleviate the workload of frontline professionals. The clinical benefits of AI extend to specialized domains, including the improved prediction of cardiovascular events and sepsis, early diagnosis of respiratory diseases, and even pandemic modeling to prevent future outbreaks [19].

While AI-assisted triage streamlines patient flow, certain discrepancies between AI decision support systems and established protocols like the MTS [27] underscore potential safety risks. Instances of misclassification in urgent scenarios highlight the imperative for rigorous clinical validation before full-scale implementation. Nevertheless, the primary efficiency of AI-guided triage lies in patient prioritization. By enabling rapid assessment in critical cases such as strokes or myocardial infarctions, these systems can reduce waiting times by up to 20%, directly translating into better clinical outcomes [34].

Studies benchmarking AI models against the Korean Triage and Acuity Scale (KTAS) have yielded significant results. For instance, Chang et al. [18] developed a model that achieved an AUC greater than 0.70 across diverse medical centers, suggesting that site-specific variations in emergency departments do not significantly hinder the model’s generalizability. In that study, Federated Learning (FL) was employed to address disparities in data quality by enabling the collaborative training of models while preserving data privacy. Because FL maintains data within each participating institution, it effectively secures sensitive information through decentralization. Notably, such systems are not intended to replace the KTAS methodology; instead, they function as Clinical Decision Support Systems (CDSS) to identify necessary revisions in initial acuity classifications. Although these tools offer robust assistance, their implementation still necessitates professional experience and clinical oversight [19].

In the context of suspected Cardiovascular Disease (CVD), specific ML-based algorithms, such as XGBoost, have reached Area Under the Curve (AUC) levels exceeding 0.90 and an accuracy of 0.78 [20]. Jiang et al. [20] advocate for AI implementation specifically to manage the high volume of low-risk visits (levels 3 and 4), which often contribute to overcrowding and inflated costs. Redirecting these low-severity populations to more appropriate healthcare tracks is a strategic recommendation to preserve emergency resources for critical cases [37].

A significant advancement in this field is the utilization of unstructured data. Kim et al. [25] and Choi et al. [19] demonstrated that incorporating textual nursing notes and voice data significantly improves a model’s ability to predict KTAS levels and identify primary symptoms. Conversely, recent evaluations of LLMs like ChatGPT show that their current performance is unsatisfactory compared to the decision-making capabilities of experienced nurses. While ChatGPT can distinguish high-acuity cases, it tends to over-classify (assigning high criticality to non-critical patients), which can be as detrimental as undertriage in a resource-constrained environment [20,26,38].

Finally, the most influential variables in these models remain physiological parameters—specifically oxygen saturation, blood pressure, heart rate, and respiratory rate—alongside biomarkers like troponin and lactate [24]. Advanced ML models excel at recognizing “faint patterns” within these variables—subtle physiological shifts that may elude human perception but indicate severe underlying pathology [23,39]. By integrating these signs with demographic data such as age and sex, AI systems can contextually adjust the definition of “normal” for each patient, ensuring a highly personalized and accurate risk estimation [21,40].

### 4.3. Limitations

The findings of this scoping review should be interpreted considering several limitations. First, there is a notable scarcity of studies specifically addressing AI applications in nurse-led triage. Most current investigations focus on medical clinical decision-making, creating a significant gap in the nursing literature. This is particularly relevant in contexts like the Brazilian healthcare system, where nurses occupy a central, autonomous role in the risk classification process.

Second, the lack of external validation across most of the identified models hinders their immediate applicability in real-world clinical settings. Without validation in diverse, multi-center environments, the reliability of these algorithms remains unproven outside their original training datasets. Furthermore, the results of this review cannot be generalized to the pediatric population, as pediatric triage was beyond the initial scope of this study.

Another challenge involves the technical complexity of the literature. Many studies employ highly specialized terminology that may be less accessible to healthcare practitioners, potentially creating a barrier to the clinical adoption of these technologies. The sheer diversity of AI methodologies—ranging from traditional ML to complex DL architectures—precluded a robust quantitative meta-analysis, limiting our ability to directly compare model performances.

Furthermore, our search strategy intentionally excluded broad terms such as ‘triage’ and ‘emergency department’ to prioritize precision over recall. Preliminary testing showed that these terms yielded an unmanageable volume of irrelevant results. Consequently, we acknowledge a potential selection bias, as studies using alternative descriptors—such as ‘risk classification’ or ‘risk assessment’—may have been omitted, potentially limiting the comprehensiveness of this review.

Finally, while the inclusion of grey literature ensured a comprehensive mapping of the field, it also introduced variability in methodological quality. As a formal risk-of-bias assessment is not mandatory for scoping reviews, our findings characterize an exploratory landscape rather than definitive clinical evidence. Consequently, the performance metrics reported herein should be interpreted with caution. These limitations emphasize the urgent need for robust, peer-reviewed clinical trials to establish the safety and efficacy of AI in emergency triage.

### 4.4. Real-world implementation challenges and explainability

Despite the promising performance metrics of AI in simulated environments (in silico), transitioning these tools into real-world clinical workflows presents formidable challenges [8,34]. A primary barrier to clinical integration is the “black box” nature of complex algorithms, particularly Deep Neural Networks [7,8]. For safe and widespread adoption, healthcare practitioners require Explainable AI frameworks that provide transparent, interpretable reasoning behind each triage output. If nurses and physicians cannot discern the logic an algorithm used to assign a specific risk level, clinical trust will remain low, potentially increasing the risk of inappropriate or delayed interventions [8,32].

Furthermore, successful implementation hinges on seamless interoperability with existing health information systems and EHRs [32,34]. Emergency departments operate in high-pressure environments that demand real-time data processing, necessitating robust IT infrastructure and high-fidelity data integration [32]. From an ethical and regulatory standpoint, the deployment of AI raises significant concerns regarding clinical accountability in cases of misclassification. There is also the persistent risk of “automation bias”, where triage nurses might over-rely on algorithmic suggestions, inadvertently suppressing their own critical clinical judgment and expertise [7,32,34].

Finally, a pervasive gap identified in the literature is the lack of external validation [8,28,34]. Most analyzed models were developed and tested using single-center datasets, suggesting that their predictive performance may degrade significantly when applied to facilities with different demographic profiles, disease prevalences, or distinct clinical workflows [8,28].

Emerging regulatory frameworks provide a foundational structure for the safe integration of artificial intelligence into clinical practice. Recent initiatives, such as the risk-based approach of the European Union Artificial Intelligence Act for high-risk healthcare systems and the U.S. Food and Drug Administration (FDA) framework for AI/ML-enabled Software as a Medical Device (SaMD), represent significant strides in governance and performance oversight [41,42]. Notably, these frameworks have advanced the discourse on adaptive algorithms, lifecycle regulation, and predetermined change control plans—elements that are particularly vital for AI systems used in emergency decision-making [41].

Despite these advances, substantial challenges persist regarding transparency, explainability, and accountability, especially for continuously learning models deployed in dynamic clinical environments [41–43]. Gaps in harmonized regulatory standards and real-world monitoring mechanisms underscore that regulatory readiness, coupled with rigorous multicenter external validation, remains a critical prerequisite. Only through these measures can AI technologies be safely integrated as reliable, complementary tools within emergency departments [41–43].

### 4.5. Final resolution

The integration of AI into risk classification represents a transformative opportunity to bolster decision-making within healthcare systems, particularly within primary care and emergency entry points. However, the findings of this review highlight a critical need to expand the current research scope. Future investigations must prioritize nursing-specific practices, the rigorous external validation of predictive models, and the adaptation of these technologies to diverse clinical environments. Cultivating interdisciplinary research—where nursing clinical expertise converges with AI innovation—is fundamental to developing tools that are safe, effective, and seamlessly integrated into daily healthcare delivery.

## 5. Conclusion

This scoping review underscores the significant potential of AI as a robust decision-support tool for risk classification and triage in emergency services. The analyzed literature reveals a broad spectrum of methodologies that demonstrate high precision in predicting clinical outcomes, while simultaneously facilitating reduced wait times and more efficient patient flow.

Despite these technological strengths, AI models should be viewed as complementary assets rather than replacements for the nuanced clinical judgment of nurses and physicians. To reach full maturity, these systems require further technological refinement, validation across heterogeneous care settings, and longitudinal analysis of their clinical impact. Ultimately, this work provides a comprehensive map of AI’s current role in triage and calls for continued interdisciplinary collaboration to ensure that its implementation enhances the safety, efficacy, and overall quality of healthcare decision-making.

## Supporting information

S1 File PRISMA-ScR Checklist. Preferred Reporting Items for Systematic reviews and Meta-Analyses extension for Scoping Reviews (PRISMA-ScR) Checklist.
(DOCX)

S2 Data Data extraction matrix. Complete dataset containing the extracted characteristics and results of the included studies.
(XLSX)
